# Effect of taurine administration on symptoms, severity, or clinical outcome of dilated cardiomyopathy and heart failure in humans: a systematic review

**DOI:** 10.12688/wellcomeopenres.17505.1

**Published:** 2022-01-10

**Authors:** Kathryn A. McGurk, Melpomeni Kasapi, James S. Ware

**Affiliations:** 1 National Heart and Lung Institute, Imperial College London, London, UK; 2 Department of Metabolism, Digestion, and Reproduction, Imperial College London, London, UK; 3 MRC London Institute of Medical Sciences, Imperial College London, London, UK; 4 Royal Brompton & Harefield Hospitals, Guy’s and St. Thomas’ NHS Foundation Trust, London, UK

**Keywords:** Taurine, DCM, Cardiomyopathy, Heart Failure

## Abstract

**Background: **Taurine, 2-aminoethanesulfonic acid, is an amino acid found in animal products. Taurine is produced for human consumption as a supplement and ingredient in beverages. Supplementation is a safe, inexpensive, and effective treatment for dilated cardiomyopathy (DCM) in domestic mammals, however it is currently unlicensed in Europe and the United States for human medical treatment. Recent genome-wide association studies of DCM have identified the locus of the taurine transporter ( *SLC6A6*). To assess whether taurine supplementation may be a novel therapeutic option for DCM, we undertook a systematic review.

**Methods: **Four electronic databases (PubMed, Cochrane Central Register, Web of Science, Biomed Central) were searched until 11/03/21. Included studies of human participants reported measured phenotypes or symptoms for cardiomyopathy, heart failure (HF), or altered left ventricle structure or function, administering taurine in any formulation, by any method. Non-English articles were excluded. Meta-analysis was completed in R software (version 3.6.0). The Newcastle-Ottawa Scale quality assessment score (NOQAS) tool was used to assess bias.

**Results: **285 articles were identified, of which eleven met our criteria for inclusion. Only one paper was deemed “high quality” using the NOQAS tool. Taurine supplementation varied across studies; by dose (500 mg to 6g per day), frequency (once to thrice daily), delivery method (tablet, capsule, drink, powder), and duration (2 to 48 weeks). Patient inclusion was all-cause HF patients with ejection fraction (EF) <50% and no study was specific to DCM. While improvements in diastolic and systolic function, exercise capacity, and haemodynamic parameters were described, only EF and stroke volume were measured in enough studies to complete a meta-analysis; the association was not significant with all-cause HF (P<0.05). No significant safety concerns were reported.

**Conclusions: **A formal clinical trial is needed to address whether taurine supplementation is beneficial to the approximately 1/250 individuals with DCM in the population.

## Introduction

Dilated cardiomyopathy (DCM) is defined by left ventricular (LV) or biventricular systolic dysfunction and dilatation not explained by abnormal loading conditions or coronary disease, and affects approximately 1 in 250 individuals ^1, 2^. Despite improvements in pharmacological and device-based therapy, clinical outcomes remain poor with a mortality of 20% at 5 years and DCM is the leading cause of heart transplantation ^3^. Clinical diagnosis of idiopathic DCM by cardiac magnetic resonance imaging includes the presence of fractional shortening <25% or ejection fraction <45%, and LV end-diastolic diameter >117%, excluding coronary disease or haemodynamic cause ^4^.

Genome-wide association studies have been recently published identifying common variants associated with risk of DCM ^5, 6^, and significant associations have been identified in the locus of the sodium- and chloride-dependent taurine transporter gene ( *SLC6A6*). *SLC6A6* encodes a multi-pass membrane protein that transports the amino acid taurine and is found at highest expression in whole blood ^7^. A mutation in the *SLC6A6* has been identified in a consanguineous family with retinal degeneration and mild hypokinetic cardiomyopathy with systolic dysfunction (shortening fraction 24–27%) and systolic dilatation of the left ventricle, which corrected with taurine supplementation after 24-months ^8^.

Taurine, or 2-aminoethanesulfonic acid, is an essential amino acid and deficiency causes photoreceptor cell degeneration. The retina and myocardium have the largest concentration of free taurine in the body ^9^. Taurine increases myocardial contractility, possibly through modulation of calcium movement and availability for excitation-contraction coupling. Taurine’s role in DCM has been well studied by veterinary medicine, and diet supplementation is a common treatment for DCM in domestic mammals ^10– 14^. It is unknown whether taurine supplementation can improve human DCM, and furthermore whether improvements are observed when taurine levels are in normal reference ranges. Taurine is currently unlicensed in the UK, EU, and US, for human medical treatment.

No existing or registered and in-progress systematic reviews have been identified on this topic. There is a need for a systematic review considering the potential health implications. This review will address whether DCM and/or heart failure (HF) symptoms and severity in humans (any patient group, population, and setting) are reduced upon administration of taurine (any dosage, delivery method, and frequency).

## Methods

The systematic review protocol has been published (Prospero ID: CRD42021241114).

### Search strategy and eligibility criteria

Four electronic databases (PubMed, Cochrane Central Register, Web of Science, and Biomed Central) were searched from inception when the review was registered on Prospero (8 ^th^ March 2021) until 11 ^th^ March 2021) with the following search strategy: (Dilated Cardiomyopathy OR DCM OR Heart failure OR Cardiomyopathy OR myocardiopathy OR Cardiac failure OR Left ventricular failure OR Left ventricular) AND (Taurine OR 2-aminoethanesulfonic acid OR 2-aminoethane-1-sulfonic acid OR Tauric acid) AND (Human OR Homo sapiens OR Patient). All included studies meet the following eligibility criteria: human studies of participants with reported measured phenotypes or symptoms for cardiomyopathy, HF, or altered left ventricle structure or function, reporting on the administration of taurine in any formulation, by any method. No restrictions were applied to geographical region or study period but articles had to be written in English.

### Study selection, data extraction and analysis

One author (K.A.M.) screened all titles and abstracts. Articles were sorted as definitely include, possibly include, and exclude. Articles classed as possibly include were reviewed by two authors (M.K., J.S.W.) to determine whether the study was eligible for inclusion. The main outcome assessed was a reduction in DCM or HF symptoms, severity or outcomes, by any measure of effect, with additional outcomes also recorded: measurements of all heart phenotypes described, any description of patient worsening, any description of side effects, any description of longitudinal/follow up outcomes.

The data was formally and unblindly extracted on pre-specified Excel (version 16.56) forms: authors, journal, publication, study design, sample size, patient characteristics, mention of additional medication intake of participants, methods of randomisation/treatment allocation, blinding, index diagnosis, taurine delivery method, dosage, frequency, and intervention duration, details of comparator or placebo, imaging characteristics assessed (if available), summary characteristics of main outcome and additional outcomes, and summary statistics of effect. Data extraction for all included studies was performed by one author (K.A.M.), with any issues requiring further clarification rectified through consultation with the other reviewing authors. Data was formally and unblindly extracted. Continuous variables were reported as mean (standard deviation) or median (range). Categorical variables were expressed as n/N (%). Summary information about the articles (e.g. aim, author overlap, main conclusion) and participant characteristics was recorded and missing or unclear information was noted as comments ( *Extended data:* Table S1).

A quantitative meta-analysis of measured phenotypes was completed if there were at least three articles describing a similar trait. Meta-analysis and forest plots were completed in R software (version 3.6.0) using the packages *meta* and *metafor*. Risk ratios for individual studies were combined using a random-effects meta-analysis. Heterogeneity was evaluated through *I* ^2^ statistic and corresponding 95% CIs. Numeric data was excluded from the review if only described in figures (found in three articles).

### Critical appraisal tool and risk of bias assessment

To assess the risk of bias in the included cohort and case-control studies, the Newcastle-Ottawa Scale quality assessment score (NOQAS) tool ^15^ was used. Using the tool, each study was judged on 8 items in three categories: selection of the study groups, comparability of groups, and the ascertainment of the outcome of interest. Studies that received 8 or 9 of a possible 9 points were regarded as high quality, whereas studies that received 6 or 7 were regarded as fair quality, and those that received 5 or less were regarded as low quality ^16^.

## Results

### Study selection and characteristics

**Figure 1.**
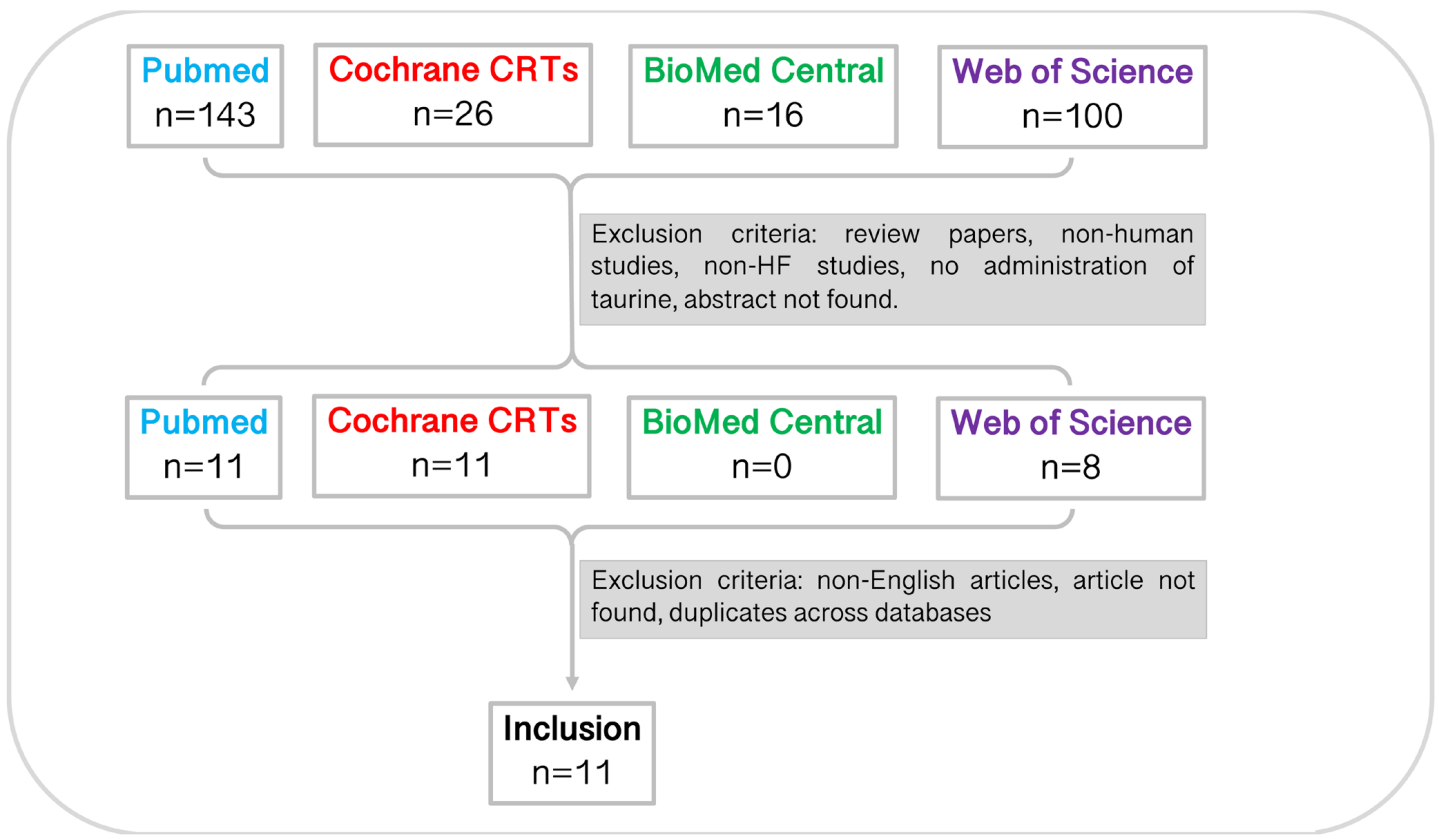
Study selection pipeline. Eleven studies were included in this systematic review. Studies were identified from each database using the specified search terms and excluded due to article type, mammal studied, disease studied, experiment undertaken, language, accessibility, and duplicates across databases. CRT, central register of controlled trials.

In total, 285 studies were identified through database searching before 11 ^th^ March 2021 (PubMed, n=143; Cochrane CRTs, n=26; BioMed Central, n=16; Web of Science, n=100). Through title and abstract screening, 30 studies remained. Following this, studies were excluded for reasons noted in Figure 1. Full-text screening and accounting for inter-database duplicates resulted in a total of eleven studies included in the systematic review. The characteristics of the studies are summarised in Table 1.

**Table 1.** Summary information on the four included studies. Echo, echocardiography; HDF, hemodiafiltration; CRP, C-reactive protein; CHF, chronic/congestive heart failure; Af, atrial fibrillation; AP, angina pectoris; HT, hypertension; X, none described; EF, ejection fraction; HF, heart failure; DCM, dilated cardiomyopathy; CAD, coronary artery disease; LVEF, left ventricular ejection fraction; METS, metabolic equivalents; MVD, mitral valve disease; NYHA, New York Heart Association functional class; CPB, cardiopulmonary bypass.

Reference	Study design: Outcome measure	Inclusion criteria (No. of patients)	Duration of follow up	Adjustment for confounders	NOQAS Score (max = 9)
Azuma *et al.* 1982	Case report: Clinical signs and symptoms of CHF and NYHA class.	CHF patients due to MVD (n=7).	4 weeks	No comparison	3
Azuma *et al.* 1985	Cohort: HF severity, electrocardiogram, phonocardiogram, carotid arterial pulse tracing, NYHA class, clinical manifestation.	CHF patients (n=14; 8 IHD, 6 valve disease).	4 weeks	Not matched or adjusted	6
Azuma *et al.* 1992	Cohort: Physical examination, resting Echo, M-mode Echo.	CHF patients admitted to cardiac rehabilitation program (n=34).	6 weeks	Statement only	6
Azuma *et al.* 1994	Cohort (long-term follow up): Self-reported and physician reported questionnaire of changes in patients’ overall condition.	CHF patients from 24 study centres (n=48).	12 months	No comparison	5
Jeejeebhoy *et al.* 2002	Cohort: NYHA class, blood count, liver and renal biochemistry, radionuclide ventriculography.	Patients scheduled for elective aortocoronary bypass surgery with CAD only and a LVEF <=40% (n=41).	30–40 days	Sex-matched	6
Beyranvand *et al.* 2011	Cohort: Exercise tolerance test (ETT), ECG, HR, BP, serum taurine measure, METS, exercise distance.	HF due to CAD with a LVEF <50% in NYHA class II or III (n=29).	2 weeks	Age-, sex-, and HF cause-matched	7
Averin *et al.* 2015	Cohort: 2D transthoracic echo, Quality of life and psychological status, Lipoproteins, cholesterol, triglycerides.	CHF patients who have undergone CPB surgery (n=48).	3 months	Age-, sex-, HF cause-matched	8
Shiohira *et al.* 2015	Case report: Echo, dry weight, and blood pressure during HDF.	HDF patients with CHF in whom dry weight was difficult to control (n=4; Af, AP, HT, X).	Varied (not further described), up to 6 months	No comparison	5
Azab *et al.* 2016	Cohort: NT­proBNP; NYHA class; Echo (not presented).	CHF patients with document EF <50% despite successive medical treatment (n=17; 9 DCM; 8 CAD).	2 months	Age- and sex- matched	7
Ahmadian *et al.* 2017 Ahmadian *et al.* 2017 (2)	Cohort: ECG, Fasting plasma taurine, troponin I, and serum lipoproteins, triglycerides, CRP, and platelet count, METS, exercise distance.	HF patients due to CAD with a LVEF <50% (n=16).	2 weeks	Age- matched	6–7 (HF cause is described in only one of the papers)

### Study quality

For the eleven studies included, quality assessment ratings were high quality (n=1; 8 points), fair (n=7; 6–7 points) and low (n=3; <5 points). This was mostly due to a lack of reporting and controlling for age and sex (comparability), lack of description of HF cause (i.e. CAD; DCM, IHD, etc.; comparability), and inadequate outcome follow up time (<3 months ^10^). Of the eleven studies included, two groups of authors wrote seven of them (63%); three studies were labelled as “Group 1” (27%) and four studies were labelled as “Group 2” (36%). In many cases across these two groups duplicate reporting of values was identified ( *Extended data:* Table S1). Of note, there were many missing values identified from these studies, mainly due to re-publication of the same cohort but reporting different phenotypes; this included sample size, blinding, medication intake, taurine delivery method, as well as basic summary statistics including age, sex, BMI, and HF aetiology.

### Article characteristics

**Figure 2.**
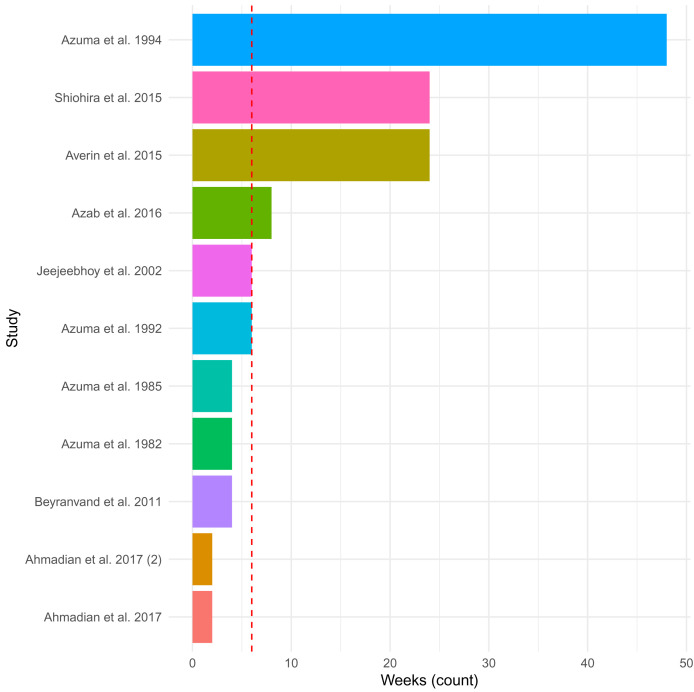
Duration of taurine administration across studies. The plot depicts the length of time in weeks of administration of taurine in the 11 included studies. The median of 6 weeks is shown as a dashed red line. Reports in month as opposed to weeks, were converted to weeks by multiplying by four.

The included eleven studies administered 500 mg to 6 g of taurine per day (in supplements provided once to thrice daily) via tablet (n=1), capsule (n=4), powder (n=1), sachet (n=1), and drink (n=1) (not described; n=3). The duration of administration ranged from 2 weeks to 12 months (median = 6 weeks; Figure 2), with the studies that assessed taurine administration at 3- and 6-months reporting only preliminary results. The most common index diagnosis for inclusion in the studies was HF patients with a left ventricular ejection fraction (LVEF) <50%, as well as patients who had undergone a particular cardiac surgery (n=2) or were not improving with current therapies (n=3). In general, the patients included in the studies had mixed aetiology of HF, mainly due to ischemic heart disease. No study was specific to DCM. The main aim of the studies was to determine the effect of taurine administration on clinical outcomes and symptoms of HF. The studies compared patients before and after administration and/or to patients on placebos (n=6 (55%); starch (n=3), carbohydrate drink (n=1), inactive placebo (n=1) or coenzyme Q10 tablet (n=1)).

### Article general findings

The conclusions of the studies describe changes in cardiac function and related parameters with taurine supplementation: increased LVEF ^17, 18^, reduction in left ventricular myocardium mass index (LVMMi) ^17^, reduction in left ventricular end-diastolic volume (LVEDV) ^19^, improved diastolic failure ^20^, improved left ventricular systolic function ^21^, decreased pre-ejection period and quotient, increased exercise capacity ^22^, and improved haemodynamic parameters (e.g., increased T wave and Q-T segment) and anti-atherogenic and anti-inflammatory effects (e.g., C-reactive protein and platelet count) ^23, 24^, and clinical course ^25, 26^.

Our prior hypothesis, based on the human genetic data, is that taurine supplementation benefits DCM specifically. There is little theoretical or experimental data to indicate whether it might impact on HF due to ischaemia or haemodynamic causes. If the effect on ischaemic and non-ischaemic cardiomyopathy differs, then this will generate heterogeneity in study results. One study of a large portion of ischemic HF patients (87%) concluded that no improvements were found in laboratory parameters or echocardiographic parameters in HF patients subjected to cardiac rehabilitation. However, the taurine treated group of patients had significant improvement in New York Heart Association Functional Classification (NYHA) (median; from 2 to 1, P = 0.002) while the control group showed improvement but without statistical significance ^27^. Patient wellbeing was examined in one of the eleven studies; mean general improvement of quality of life and wellbeing in patients with HF was 22.6 % compared to 16.6% in the placebo arm, with increased improvement among women ^17^. 

### Article safety findings

Safety data was described in detail in one study of taurine as part of a multivitamin and mineral supplement drink ^19^. While these adverse effects may be due to taurine supplementation, the supplementation consisted of many vitamins and minerals, so it is unlikely that taurine had a causal role in the adverse reactions. One patient (5%) in the supplement group had nausea and another had a single episode of vomiting. Two patients developed diarrhoea and one of them dropped out of the study as a result. The administration of supplement compared with placebo did not influence blood biochemistry (ALP, ALT, AST), nor blood urea nitrogen, except for significantly higher creatinine levels at the preoperative assessment. One patient from the treatment and control groups had myocardial infarction. One patient in the supplement group developed pneumonia and had a 6-day stay in the intensive care unit. Three patients developed clinically significant renal failure, one patient had renal artery stenosis, one patient had renal failure related to diabetes, and the final patient had multiple medical problems after the operation with a wound infection, urinary tract infection, and atrial fibrillation. One patient died during the study having developed coagulopathy after an operation. Two patients dropped out of the study in the supplement group, one after developing diarrhoea and the other dropped out after developing renal failure caused by renal artery stenosis. A further study reported that one patient in the supplementation group died of hepatoma ^25^.

### Focussed analysis on long-term taurine treatment

A preliminary report of a long-term study of taurine administration in 103 chronic HF patients added to conventional therapy described that after three months, 60% of the taurine group were subjectively considered by investigators to have symptomatic improvement compared to 20% in the non-taurine group (no placebo was used in the study). After one year, 30% of patients reported that they felt moderately better with 44% feeling slightly better compared to just 24% of the non-taurine group feeling slightly better. At the same timepoint, 39% of patients on taurine had better breathlessness on exertion, while only 15% in the non-taurine group had this decreased breathlessness alongside 10% worsening. Lack of energy and swelling in ankles or legs improved less frequently and deteriorated more in patients in the non-taurine group ^25^. This was the only study assessing long-term taurine supplementation.

### The effect of taurine administration on DCM and heart failure

**Figure 3.**
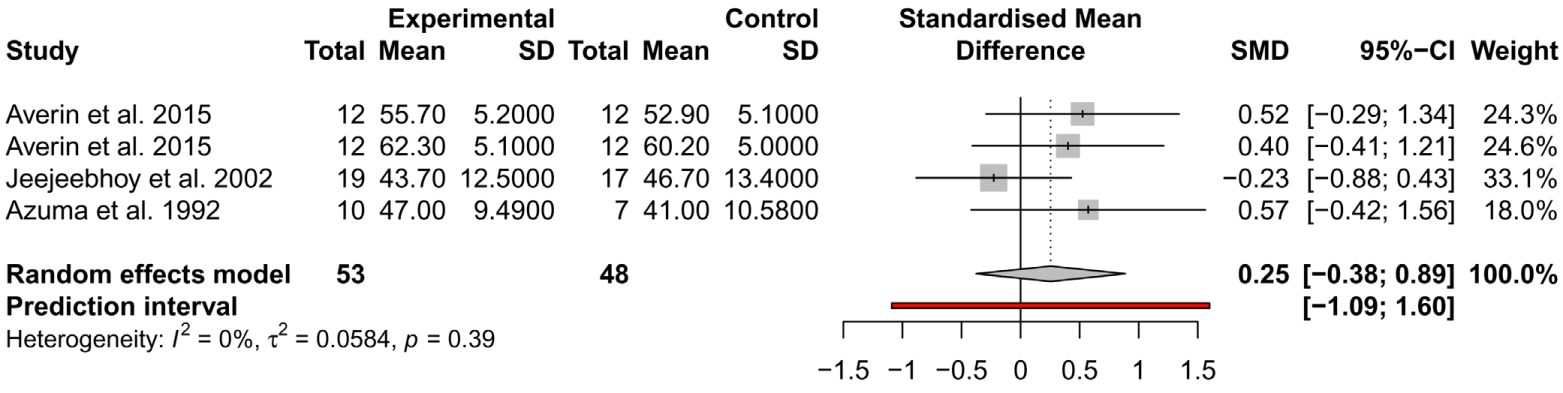
Assessment of the effect of taurine supplementation on ejection fraction (%) in all-cause HF patients. The plot depicts a trend to increase EF, described from three studies (two cohorts from one study). Taurine supplementation was not significant in altering ejection fraction in all-cause HF patients.

**Figure 4.**
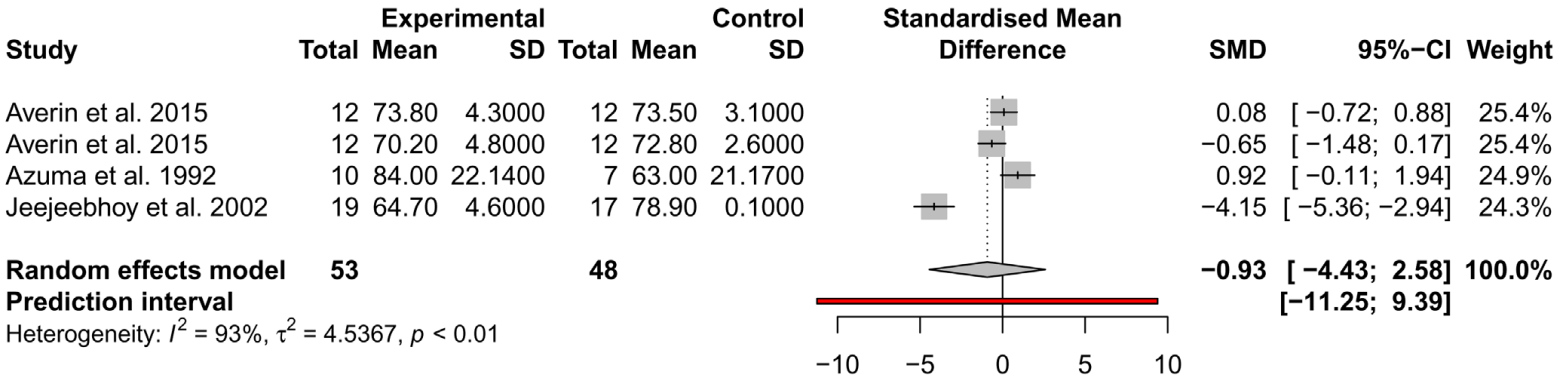
Assessment of the effect of taurine supplementation on stroke volume (ml) in all-cause HF patients. The plot depicts a trend to reduce SV, described from three studies (two cohorts from one study). Taurine supplementation was not significant in altering stroke volume in all-cause HF patients.

While many traits were examined for alteration by taurine administration, such as imaging and exercise ( *Extended data:* Table S1), only ejection fraction (EF), stroke volume (SV), and NYHA were analysed across at least three studies from different authors and could be included in a meta-analysis. EF and SV were not significantly different across three studies, with the analysis of SV showing high heterogeneity ( *I* ^2^ = 93%) ( Figure 3 and Figure 4). This may be due to the small sample size of the studies and the inclusion of mixed heart failure aetiologies. NYHA class was described to improve in two studies by the same author; for 5/7 patients (71%), an improvement to NYHA class II after 4 weeks of taurine continued as long as taurine was continued for 3 to 12 months ^25^; and improvement in NYHA class was observed in 4/14 patients (29%) administered taurine without worsening, compared to no patients improving on the placebo alongside 2/14 worsening in NYHA class (14%) ^18^.

DCM has been defined by the presence of fractional shortening <25% or EF <45%, and LV end-diastolic diameter >117% ^4^. There were only two studies that assessed the effect of taurine administration on left ventricular diastolic diameter; one was not significant ^17^ and another described left ventricular diastolic “dimension”, presumably in millilitres (units were not specified), that reduced with supplementation in four individuals from a mean of 51 (+/- 13) to 44 (+/- 11) ^20^. End diastolic volume (EDV) was assessed in two studies; in one (n=4), supplementation nominally reduced mean LVEDV from 131 ml (+/- 75 ml) to 94 ml (+/- 58 ml) ^20^, and in the other (n=38), supplementation was associated with significantly reduced EDV from 171 ml (+/- 50 ml) to 159 ml (+/- 51 ml) ^19^. Further assessment of these parameters in a DCM-specific cohort would aid our understanding of the influence of taurine supplementation on the heart.

## Discussion

The aim of the systematic review was to identify the current literature describing the effects of taurine on DCM and HF in humans and assess whether it may be a novel therapeutic option for patients. This review identified eleven studies that met criteria for inclusion but were not specific to DCM patients. Inclusion criteria to the studies was for all-cause heart failure patients with EF <50%. Only one paper was deemed high quality using the NOQAS assessment tool, therefore, 91% of included studies were “fair” or less in quality. Seven of the eleven articles (63%) were published by two groups of researchers; in many cases the articles shared summary information and missing values. Administration of taurine supplements varied across all studies; by dose (500 mg to 6g per day), by frequency of supplementation (once to thrice daily), by delivery method (tablet, capsule, drink, powder), and by duration of the supplementation (2 to 48 weeks). Only 55% of studies included a placebo group when assessing the effects of taurine supplementation.

While many studies reported significant improvements in LVEF, LVEDV, LVMMi, exercise capacity and haemodynamic parameters, we could only include two parameters in a meta-analysis of three placebo-controlled studies; EF and SV, which were both not significant. Thus, most studies assessed LV function, but not chamber dimensions which are essential to the diagnosis of DCM.

Taurine supplementation had few safety concerns reported in the studies ^19^. As we cannot conclude whether taurine supplementation is beneficial to patients with DCM from this systematic review, we plan to undertake a formal clinical trial to address this, and to investigate whether improvement is only observed in taurine deficient patients.

There were several limitations to the review process. The exclusion of non-English articles, and pre-print articles that are not included in the databases searched, may have excluded articles that would otherwise reach inclusion criteria. In addition, there are reported limitations to using the NOQAS tool that have been discussed elsewhere ^28, 29^.

## Conclusion

Taurine supplementation as a treatment for dilated cardiomyopathy would be a much needed and cost-effective addition to current therapeutics for DCM patients. This review cannot conclude whether taurine supplementation is beneficial to DCM patients, as current human studies include small sample sizes with heterogeneous patient inclusion for all-cause heart failure. A clinical trial is required to assess whether taurine supplementation is beneficial to patients with DCM.

## Data availability

### Underlying data

All data underlying the results are available as part of the article and no additional source data are required.

### Extended data

Zenodo: kmcgurk/TaurineDCM: First release, https://doi.org/10.5281/zenodo.5785673 ^30^

This project contains the following extended data:

- Table S1: Data extracted from each of the eleven studies included in the review paper.

### Reporting guidelines

Zenodo: PRISMA checklist for ‘Effect of taurine administration on symptoms, severity, or clinical outcome of dilated cardiomyopathy and heart failure in humans: a systematic review’, https://doi.org/10.5281/zenodo.5785673 ^30^

Data are available under the terms of the Creative Commons Zero "No rights reserved" data waiver (CC0 1.0 Public domain dedication).
