# Supplementary material for: Effect of taurine administration on symptoms, severity, or clinical outcome of dilated cardiomyopathy and heart failure in humans: a systematic review
Source: Wellcome Open Res. 2022 Jul 7;7:9. Originally published 2022 Jan 10. [Version 3] doi: 10.12688/wellcomeopenres.17505.3 (PMC9257265; doi:10.12688/wellcomeopenres.17505.3)
Supplement: Peer review history

# Peer review: Reviewer response for version 2

**DOI:** 10.21956/wellcomeopenres.19584.r51169

**Published:** 2022-07-05

**Authors:** Alexandra Butters, Joanna Sweeting

**Affiliations:** 1 Centre for Population Genomics, Garvan Institute of Medical Research and University of New South Wales, Sydney, Australia; 2 Centre for Population Genomics, Murdoch Children’s Research Institute, Melbourne, Australia; 3 Faculty of Medicine and Health, The University of Sydney, Sydney, Australia

The authors report a systematic review to address whether DCM and/or heart failure symptom severity in humans is reduced upon administration of taurine. They identified 285 articles, of which eleven met the criteria for inclusion (phenotypes or symptoms of cardiomyopathy, heart failure, altered LV ventricle structure or function and the reporting of administration of taurine by any method). Only 1 paper was considered high quality. Taurine supplementation varied by dose, frequency, and duration; none of the papers were specific to DCM. Only EF and stroke volume were available from enough studies to complete a meta-analysis; no association with all-cause HF was found (P<0.05) and no significant safety concerns were reported. The authors conclude that the review is unable to conclude whether taurine supplementation is beneficial to DCM patients and suggest a clinical trial is required. The manuscript is clearly written and represents novel work.

 The manuscript could benefit from the following:

- Addition of a table showing the reason why each study was not included and/or number excluded per criteria e.g. number non-English articles, articles not found etc (Figure 1).

Are the rationale for, and objectives of, the Systematic Review clearly stated?

Yes

Is the statistical analysis and its interpretation appropriate?

Yes

Are sufficient details of the methods and analysis provided to allow replication by others?

Yes

Are the conclusions drawn adequately supported by the results presented in the review?

Yes

Reviewer Expertise:

Inherited heart disease, sudden cardiac death, epidemiology, biostatistics, environmental sciences

We confirm that we have read this submission and believe that we have an appropriate level of expertise to confirm that it is of an acceptable scientific standard.

# Response

**Published:** 2022-07-05

**Authors:** Kathryn McGurk

**Affiliations:** Imperial College London, UK

We thank Alexandra Butters and Doctor Joanna Sweeting for taking the time to review this article and for their summary statements.  

 We have added the table to the manuscript, as they suggested.

# Peer review: Reviewer response for version 2

**DOI:** 10.21956/wellcomeopenres.19584.r48537

**Published:** 2022-03-07

**Authors:** Abdallah Al-Mohammad

**Affiliations:** 1 Department of Infection, Immunity & Cardiovascular Disease, University of Sheffield, Sheffield, UK; 2 Cardio-thoracic Directorate, Sheffield Teaching Hospital NHS Foundation Trust, Sheffield, UK

In their systemic review of the effects of taurine administration in patients with either dilated cardiomyopathy or indeed other types of heart failure, McGurk *et al.* attempted to learn the impact of taurine supplementation on the symptoms, severity, or clinical outcome of patients with these syndromes. The authors were confronted with relative paucity of data specific to dilated cardiomyopathy (DCM). The basic scientific principles underpinning their interest in the topic, however, stem from an observation associating risk of development of DCM to a defect in the locus of the sodium- and chloride-dependent taurine transporter gene (SLC6A6).

 The authors accepted studies that ranged from case reports, with a loose definition of the syndrome in question including patients whose heart failure was related to ischaemic heart disease. The latter group of patients are unlikely to have a common base with DCM. This approach while justified on the basis that taurine was administered in these studies, is less likely to lead to reliable answers to the questions posed.

 The authors reported one of the weaknesses of the studies reviewed: lack of description of the heart failure cause. This is a major problem if we were to use the data thus collected to make statements about the role of taurine and its deficiency in the pathogenesis or aetiology of dilated cardiomyopathy. Indeed, no study was specific for DCM. The authors correctly stated that no conclusion about the potential benefit of taurine in DCM patients could be reached.

 Although they included eleven studies, there was duplication of reporting of cases as 63% of the papers came from two groups. Moreover, the improvement in the patients’ well-being was only tested in one of the 11 studies. This diminishes the reliability of the systemic review’s ability to assess the impact of taurine supplementation on the symptoms or the outcome. In addition, only 55% of the studies included a placebo group. The latter further minimises the reliability of the results of the remaining studies in informing us about any potential impact of the proposed therapy.

 The authors looked at parameters such as ejection fraction and stroke volume and concluded effectively that there is no demonstrable impact of taurine on the outcome. I do not think that the absence of positive results here necessarily provides us with more than a methodological exercise.

 When one is yet to be certain about the aetiological impact of a genetically determined defect, one should concentrate the efforts on the subset of patients certain to have that defect, before attempting to test the therapeutic impact of the intervention.

 Notwithstanding those caveats, we remain totally uncertain whether the effects of taurine supplementation would be detectable after brief courses of therapy.

 I support the authors in concluding that a clinical trial is needed to assess whether taurine supplementation is beneficial for patients with DCM. They are also correct that such a study needs to address whether the benefit of taurine would be limited to taurine-deficient patients or not.

 In addition, It would be vital to assess in a cohort of patients with DCM, the incidence of taurine deficiency, the incidence of the genetically determined deficiency, and then to study the impact of taurine supplementation on symptoms, quality of life, imaging, and haemodynamic parameters before assessing the impact of that therapy on outcomes such as hospitalisation or survival.

Are the rationale for, and objectives of, the Systematic Review clearly stated?

Yes

Is the statistical analysis and its interpretation appropriate?

Yes

Are sufficient details of the methods and analysis provided to allow replication by others?

Yes

Are the conclusions drawn adequately supported by the results presented in the review?

Partly

Reviewer Expertise:

Heart failure epidemiology and therapeutics. Cardiac imaging. Infective endocarditis.

I confirm that I have read this submission and believe that I have an appropriate level of expertise to confirm that it is of an acceptable scientific standard.

# Peer review: Reviewer response for version 1

**DOI:** 10.21956/wellcomeopenres.19355.r47935

**Published:** 2022-01-24

**Authors:** Abdallah Al-Mohammad

**Affiliations:** 1 Department of Infection, Immunity & Cardiovascular Disease, University of Sheffield, Sheffield, UK; 2 Cardio-thoracic Directorate, Sheffield Teaching Hospital NHS Foundation Trust, Sheffield, UK

In their systemic review of the effects of taurine administration in patients with either dilated cardiomyopathy or indeed other types of heart failure, McGurk et al. attempted to learn the impact of taurine supplementation on the symptoms, severity or clinical outcome of patients with these syndromes. The authors were confronted with relative paucity of data specific to dilated cardiomyopathy (DCM), even though the basic scientific principles underpinning their interest in the topic stem from an observation related to risk of DCM being associated to a defect in the locus of the sodium- and chloride-dependent taurine transporter gene (SLC6A6).

 The authors accepted studies that ranged from case reports, with loose definition of the syndrome in question including patients whose heart failure was related to ischaemic heart disease. The latter group of patients are unlikely to have a common base with DCM. This approach while justified on the basis that taurine was administered in these studies, is less likely to lead to reliable answers to the questions posed.

 The authors reported as one of the weaknesses of the studies reviewed: lack of description of the heart failure cause. This is a major problem if we were to use the data thus collected to make statements about the role of taurine and its deficiency in the pathogenesis/aetiology of dilated cardiomyopathy. Indeed, no study was specific for DCM. The authors correctly concluded that no conclusion about the potential benefit of taurine in DCM patients could be reached.

 Although they included eleven studies, there was duplication of reporting of cases as 63% of the papers came from two groups. It is noticeable that the improvement in the patients’ well-being was only tested in one of the 11 studies. This diminishes the reliability of the systemic review’s ability to assess the impact of taurine supplementation on the symptoms or the outcome. In addition, only 55% of the studies included placebo group. The latter further minimises the reliability of the results of the remaining studies in informing us about any potential impact of the proposed therapy.

 The authors looked at parameters such as ejection fraction and stroke volume and concluded effectively that there is no demonstrable impact of taurine on the outcome. I personally do not think that the absence of positive results in here necessarily provides us with more than a methodological exercise. 

 When one is yet to be certain about the aetiological impact of a genetically determined defect, one should concentrate the efforts on the subset of patients certain to have that defect, before attempting to test the therapeutic impact of the intervention.

 Notwithstanding those caveats, we remain totally uncertain whether the effects of taurine supplementation would be detectable after brief courses of therapy.

 I would not agree with the first part of the conclusion by the authors about taurine being expected to be a much needed therapy and that it would be cost effective for patients with DCM. Having said that, I support the last part of their conclusion that a clinical trial is needed to assess whether taurine supplementation is beneficial for patients with DCM. They are also correct that such a study needs to address whether the benefit of taurine would be limited to taurine-deficient patients or not.

 It would, however, be vital to assess in a cohort of patients with DCM, the incidence of taurine deficiency, the incidence of the genetically determined deficiency and then to study the impact of taurine supplementation on symptoms, quality of life, imaging and haemodynamic parameters before assessing the impact of that therapy on outcomes such as hospitalisation or survival.

 Their manuscript requires some corrections:

- In the introduction, paragraph 1, the cMR definition of DCM includes (LV end-diastolic diameter >117%). The authors are advised to add that this refers to 117% of the upper normal range.
- In the introduction, paragraph 2, the authors stated (which corrected with taurine supplementation after 24-months). This should be corrected into (which was corrected with taurine supplementation after 24-months).
- In the introduction, paragraph 3, the authors stated (Taurine, or 2-aminoethanesulfonic acid, is an essential amino acid and deficiency causes photoreceptor cell degeneration.) This should be corrected to become: (Taurine, or 2-aminoethanesulfonic acid, is an essential amino acid and its deficiency causes photoreceptor cell degeneration.)
- In the methods, in the search strategy, the terms used included at the end [OR Left ventricular]. There is clearly a word missing here and they need to clarify the missing word there.

Are the rationale for, and objectives of, the Systematic Review clearly stated?

Yes

Is the statistical analysis and its interpretation appropriate?

Yes

Are sufficient details of the methods and analysis provided to allow replication by others?

Yes

Are the conclusions drawn adequately supported by the results presented in the review?

Partly

Reviewer Expertise:

Heart failure epidemiology and therapeutics. Cardiac imaging. Infective endocarditis.

I confirm that I have read this submission and believe that I have an appropriate level of expertise to confirm that it is of an acceptable scientific standard, however I have significant reservations, as outlined above.

# Response

**Published:** 2022-02-02

**Authors:** Kathryn McGurk

**Affiliations:** Imperial College London, UK

We thank Professor Al-Mohammad for his detailed review of this article and for his summary statements.

 We agree with the reviewer and have removed the first sentence from the conclusion.

 We agree with the reviewer about the need to assess the incidence of taurine deficiency in DCM.

 We have corrected the DCM definition in the Introduction and Results by adding “of the upper normal range”.

 We have corrected the Introduction by adding “was”.

 We have corrected the Introduction by adding “its”.

 We kept the search strategy terms broad to identify any article reporting on left ventricular alteration, thus the search term “left ventricular *” was used. This has been altered in text.
